# Clostridioides difficile Infection Dysregulates Brain Dopamine Metabolism

**DOI:** 10.1128/spectrum.00073-22

**Published:** 2022-03-24

**Authors:** Akhil A. Vinithakumari, Piyush Padhi, Belen Hernandez, Susanne Je-Han Lin, Aaron Dunkerson-Kurzhumov, Lucas Showman, Matthew Breitzman, Caroline Stokes, Yousuf Sulaiman, Chandra Tangudu, Deepa A. Kuttappan, Muhammed S. Muyyarikkandy, Auriel A. Willette, Gregory J. Phillips, Vellareddy Anantharam, Ann Perera, Brett A. Sponseller, Anumantha Kanthasamy, Shankumar Mooyottu

**Affiliations:** a Department of Veterinary Pathology, Iowa State University, Ames, Iowa, USA; b Department of Biomedical Sciences, Iowa State University, Ames, Iowa, USA; c W. M. Keck Metabolomics Research Laboratory, Iowa State University, Ames, Iowa, USA; d Department of Veterinary Microbiology and Preventive Medicine, Iowa State University, Ames, Iowa, USA; e Department of Animal Science, University of Connecticut, Storrs, Connecticut, USA; f Department of Food Science and Human Nutrition, Iowa State University, Ames, Iowa, USA; g Department of Physiology and Pharmacology, University of Georgia, Athens, Georgia, USA; Johns Hopkins Hospital

**Keywords:** gut-brain axis, *Clostridioides difficile*, p-cresol, dopamine

## Abstract

Gastrointestinal illnesses and dysbiosis are among the most common comorbidities reported in patients with neurodevelopmental disorders. The manuscript reports that C. difficile infection (CDI), predisposed by antibiotic-induced gut dysbiosis, causes significant alterations in dopamine metabolism in major dopaminergic brain regions in mice (*P* < 0.05). In addition, C. difficile infected mice exhibited significantly reduced dopamine beta-hydroxylase (DBH) activity compared to controls (*P* < 0.01). Moreover, a significantly increased serum concentration of p-cresol, a DBH inhibiting gut metabolite produced by C. difficile, was also observed in C. difficile infected mice (*P* < 0.05). Therefore, this study suggests a potential mechanistic link between CDI and alterations in the brain dopaminergic axis. Such alterations may plausibly influence the precipitation and aggravation of dopamine dysmetabolism-associated neurologic diseases in infected patients.

**IMPORTANCE** The gut-brain axis is thought to play a significant role in the development and manifestation of neurologic diseases. This study reports significant alterations in the brain dopamine metabolism in mice infected with C. difficile, an important pathogen that overgrows in the gut after prolonged antibiotic therapy. Such alterations in specific brain regions may have an effect on the precipitation or manifestation of neurodevelopmental disorders in humans.

## INTRODUCTION

Although the involvement of the gut-brain axis is increasingly implicated in neurological disorders, mechanistic evidence that connects gut bacteria, gut metabolites, and neuronal dysregulation is sparse (1). Disruption of the normal gut microbiota is known to cause an overgrowth of unfavorable bacterial communities that increase toxic metabolites in the gut (2). Several of these metabolites are known to impact neurotransmission and brain functions, underscoring the importance of the gut-brain axis in health and disease (3).

Gut dysbiosis induced by prolonged antibiotic therapy is the most common cause of *Clostridiodes difficile* infection and associated diarrhea in infants and adults (4, 5). Human C. difficile strains produce a significant amount of p-cresol, unlike most other gut bacteria (6–8). C. difficile produces 10 to 1000 times more p-cresol than other known p-cresol producing bacteria in the gut (9, 10). Indeed, recent studies demonstrated p-cresol induced behavioral alterations in mice by dysregulating the dopaminergic axis (11, 12). What remains unknown specifically is the functional consequence of C. difficile infection on the dopaminergic neurotransmission from a gut-brain axis standpoint. Moreover, the involvement of possible mediators, such as dopamine beta-hydroxylase (DBH), that connects gut microbial p-cresol to dopamine (DA) dysmetabolism needs to be demonstrated. Thus, the current work investigates the effect of antibiotic-induced C. difficile infection on the DA metabolism in the brain.

## RESULTS AND DISCUSSION

### Antibiotic induced gut dysbiosis and C. difficile infection in mice.

**FIG 1:**
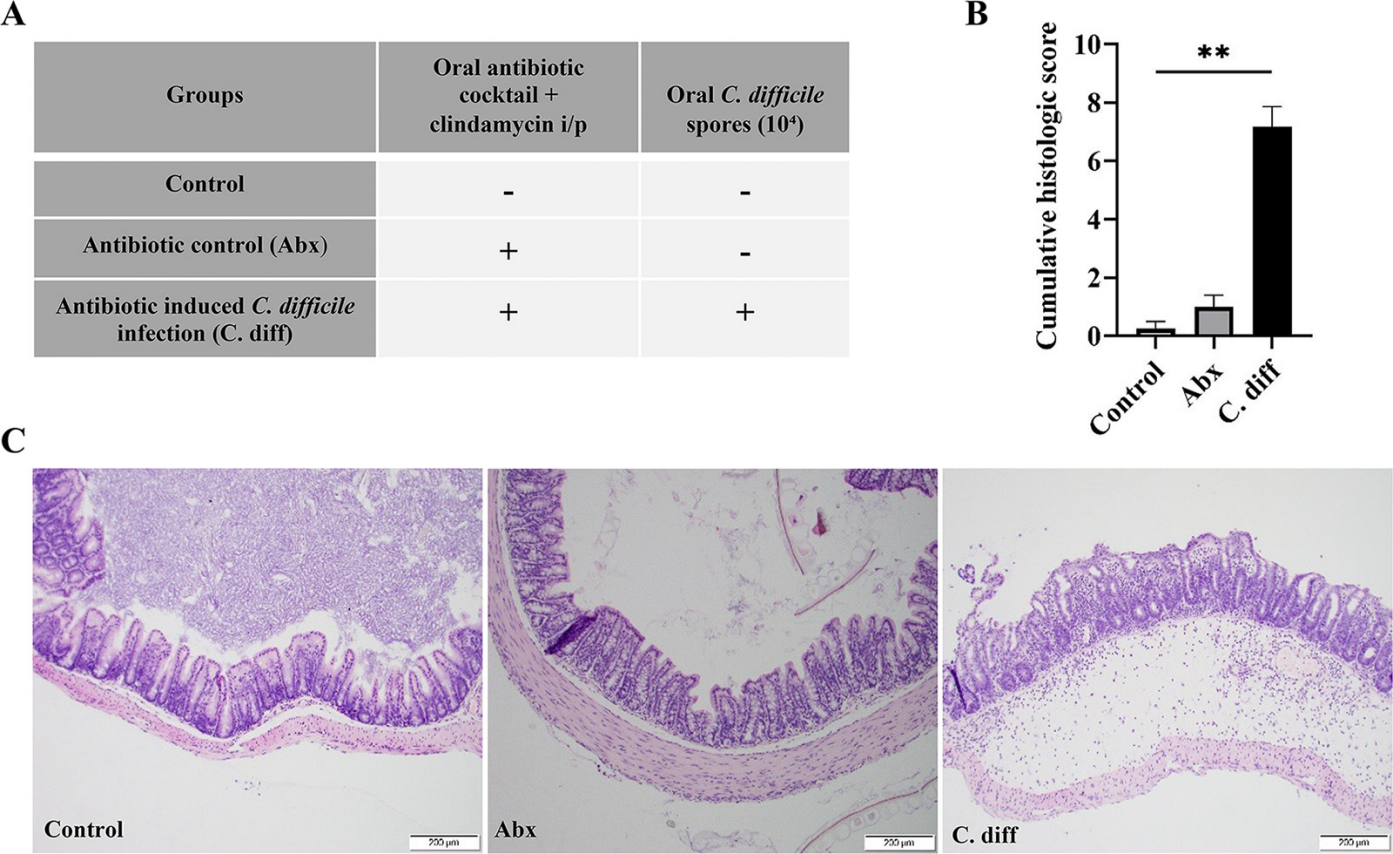
Alterations in the gut mucosa in antibiotic-induced and C. difficile infected mice. (A) Experimental outline: Three-4 weeks old C57BL/6 mice (*n* = 18/group) are treated with an oral antibiotic cocktail or PBS and an intraperitoneal clindamycin injection or PBS to induce gut dysbiosis and then challenged with 10^4^ C. difficile spores or PBS. The serum, cecal contents, and tissues were collected 2 days postinfection (*n* = 18). (B) Histopathology of the colon from different treatment groups: colonic mucosa of the C. diff group exhibited severe epithelial damage, mucosal edema, and neutrophil infiltration compared to controls. (C) Cumulative histologic scores (0–9 scale): C. difficile infection induces severe colitis in mice (*n* = 8). All data (C) are presented as the mean, and error bars indicate SEM. One-way ANOVA with adjusted *P*-value was used to test for statistical significance **P < 0.01, *P < 0.05.

**FIG 2:**
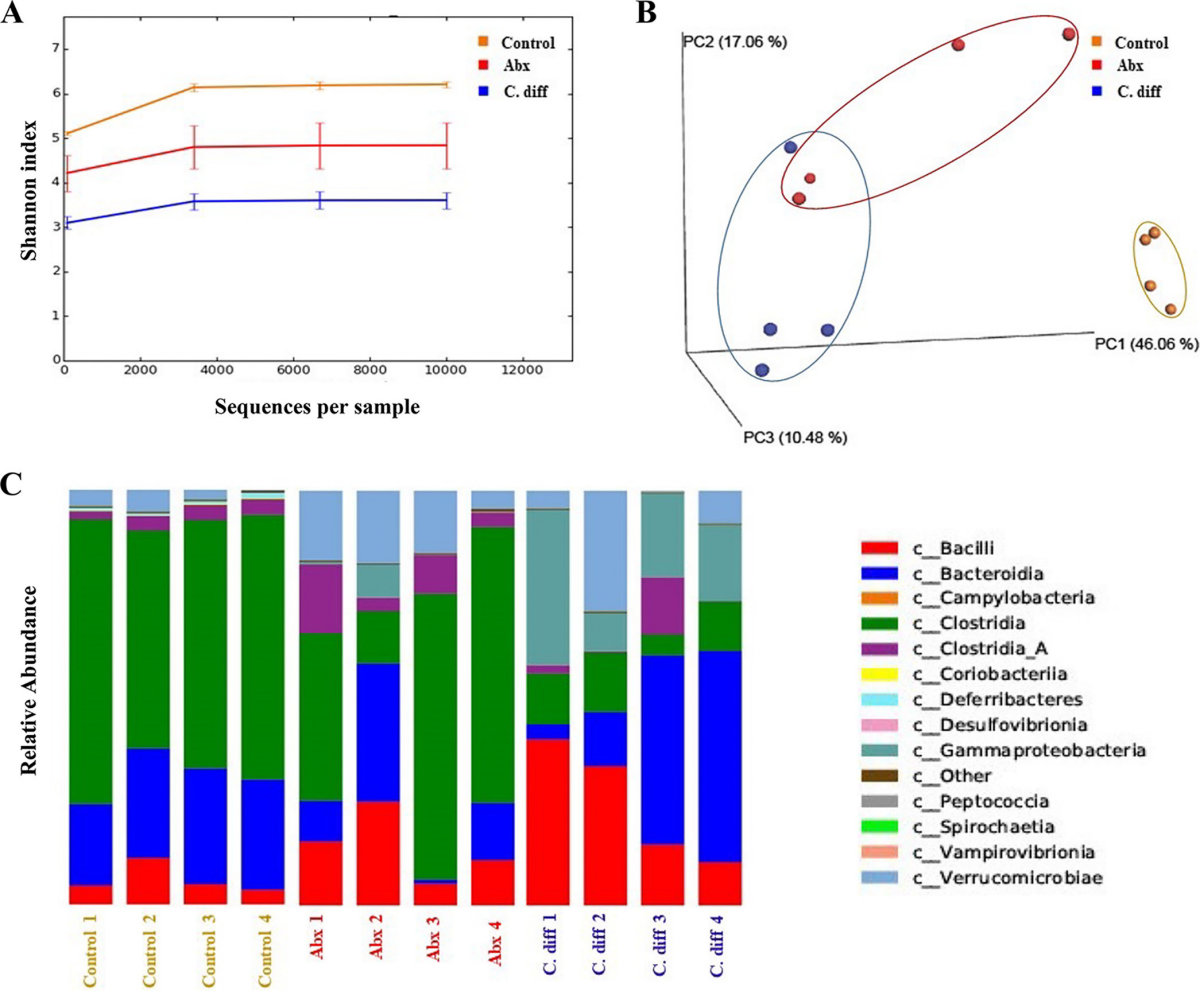
Antibiotic treatment and C. difficile infection induced gut-dysbiosis in mice. (A) Alpha diversity plot of different treatment groups: antibiotic treatment and C. difficile infection reduced the alpha diversity of the gut microbiome as indicated by a lower Shannon index than control. (*n* = 4). (B) Principal coordinate analysis (PCoA) plot for beta diversity (weighted Bray-Curtis distances): both Abx and C. diff groups clustered away from control mice in terms of gut microbiota beta diversity (*n* = 4). (C) The relative abundance of bacteria at class level: antibiotic treatment and C. difficile infection altered the abundance of different major bacterial communities in mice (*n* = 4).

To determine CDI induced alterations in the dopaminergic axis, we performed a C. difficile challenge study in mice following a published protocol (13). For this experiment, 3-week-old C57BL/6 mice were administered an oral antibiotic cocktail followed by an intraperitoneal clindamycin injection to induce gut dysbiosis. Mice were then challenged with approximately 10^4^ C. difficile UK1 spores. From the previous experiments conducted by the authors and other investigators, it is known that 10^4^ C. difficile UK1 spores reliably induce clinical CDI with less mortality in mice compared to high dose (10^6^ to 10^7^ spores) challenge, simulating sublethal or persistent CDI in humans (13, 14). Diarrhea, or wet tail, was noticed in all C. difficile challenged mice (C. diff group) on day-2 postinoculation. As expected, the colon of the mice challenged with 10^4^ C. difficile UK1 (C. diff) exhibited notable pathological changes in the mucosal epithelium, as evidenced by necrosis, moderate neutrophilic inflammatory infiltrates in the lamina propria and submucosa, and marked submucosal edema, contributing to a significantly increased cumulative colitis score (Fig. 1A to C). Gut dysbiosis in mice with only antibiotic treatment (Abx) and C. diff groups was confirmed by demonstrating a reduced diversity of the gut microbiota and altered abundance of various bacterial communities compatible with previously published findings (Fig. 2A–C) (15, 16).

### C. difficile induced alterations in catecholamine profiles in striatum and prefrontal cortex.

Further, catecholamine profiles of DA and its metabolites were assessed in different mouse brain regions relevant to dopaminergic neurotransmission. Two major dopaminergic pathways in the brain significant to autism spectrum disorders (ASD) include the mesocorticolimbic circuit and the nigrostriatal circuit (17). Substantia nigra and the ventral tegmental area are the two major DA producing areas in the brain. Neurons from the substantia nigra project to the dorsal striatum, forming the nigrostriatal circuit, which is associated with the motor aspects of goal-directed behavior. The ventral tegmental area projects to the prefrontal cortex and the ventral striatum forming the mesocorticolimbic circuit, which is involved in reward and motivation-related behavior.

**FIG 3:**
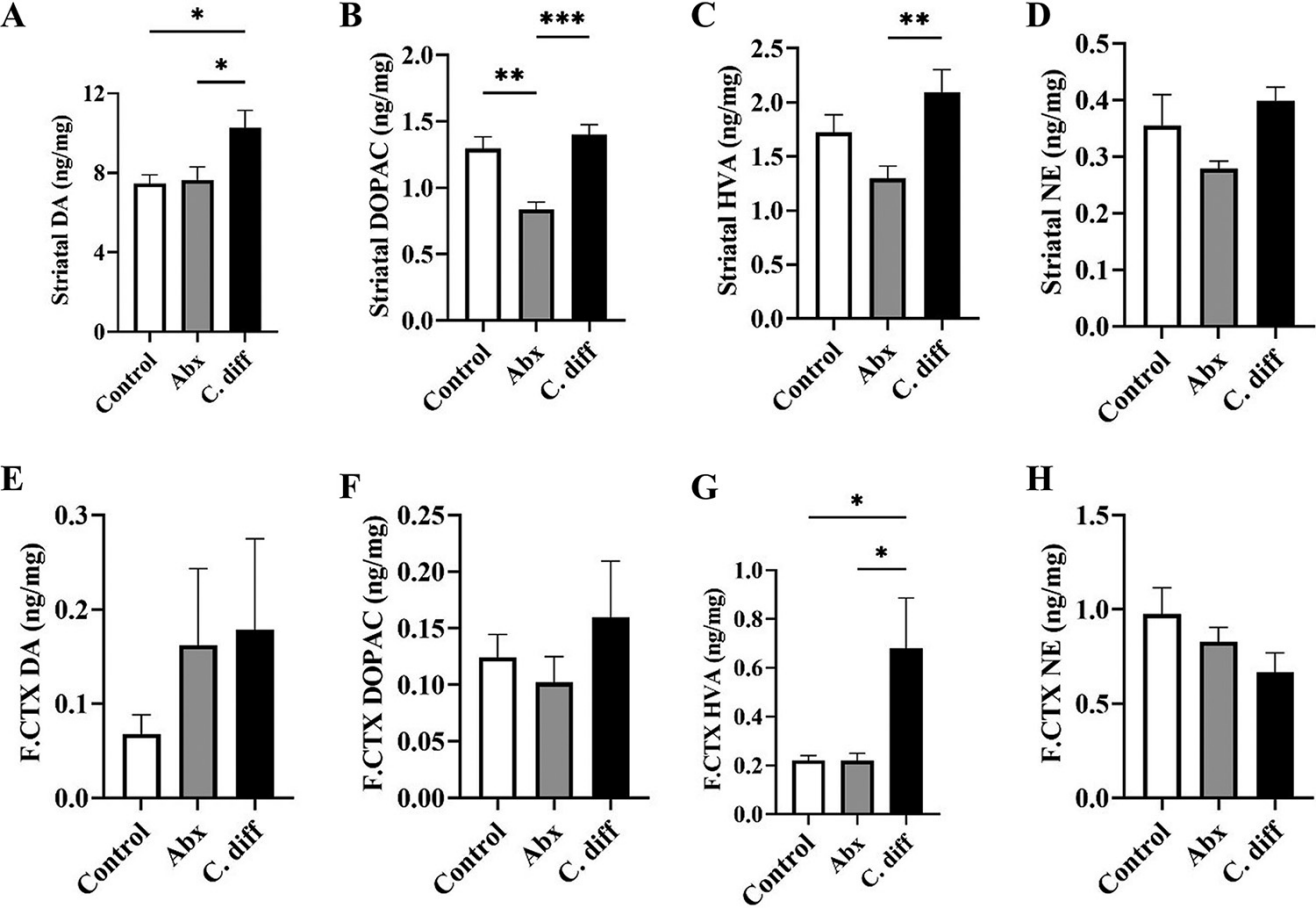
Alterations in striatal and prefrontal cortex catecholamine profiles in antibiotic treated and C. difficile infected mice. Catecholamine concentrations in striatal and prefrontal cortex brain regions of control, antibiotic control (Abx), and C. difficile challenged (C. diff) mice at 2 days postinoculation was quantitated by the HPLC-ED method. Striatal catecholamine concentration in different treatment groups: (A) dopamine (DA), (B) its metabolite 3, 4-dihydroxyphenylacetic acid (DOPAC), (C) homovanillic acid (HVA), and (D) norepinephrine (NE) levels; prefrontal cortex (F.CTX) catecholamine concentration in different treatment groups: (E) dopamine (DA), (F) its metabolite 3,4-dihydroxyphenylacetic acid (DOPAC), (G) homovanillic acid (HVA), and (H) norepinephrine (NE) levels. *n* = 5. All data (A–H) are presented as the mean, and error bars indicate SEM. One-way ANOVA with adjusted *P*-value was used to test for statistical significance **P < 0.01, *P < 0.05.

The most notable observation made in this study is a significantly increased DA in the striatum (Fig. 3A). A significantly increased striatal DA concentration was observed in the C. diff group compared to the untreated control (*P* = 0.0360). However, concentrations of DA metabolic products 3,4-dihydroxyphenylacetic acid (DOPAC) and homovanillic acid (HVA) did not significantly differ from that of control animals (Fig. 3B and C). Similarly, no significant difference was observed in the norepinephrine (NE) concentration in the striatum of C. difficile challenged mice compared to the negative-control group (Fig. 3D). Striatal DA concentrations were unaltered or slightly reduced in the antibiotic control group (Abx) compared to the untreated control (Fig. 3A). In contrast, significantly lower striatal DOPAC (*P* = 0.0010) and HVA concentrations (*P* = 0.0094) were observed in the Abx group compared to the negative control (Fig. 3B–C). No statistically significant difference was observed in the striatal NE levels in Abx (Fig. 3D). In the prefrontal cortex, a significant increase in HVA was observed in the C. difficile challenged mice compared to both negative (*P* = 0.0158) and antibiotic control groups (*P* = 0.0191) (Fig. 3G).

Dopamine release in the striatum and prefrontal cortex is generally associated with reward response, motivation, pleasure, and addiction. In contrast, an increase in DA levels in these brain regions in response to a persistent painful condition (CDI) is unusual, as pain is a significant factor that ought to be considered in a CDI study. Generally, DA is considered anti-nociceptive, and a persistent noxious stimulus is often associated with decreased DA levels in the brain (18). Therefore, a significantly increased DA and/or its degradation products observed in the striatum and prefrontal cortex could be potentially attributed to a pathway that directly alters DA metabolism. Increased DA (or its degradation products) to NE ratio has been implicated in disorders of DBH activity, such as low DBH levels due to mutations in DBH coding genes (19, 20). Therefore, inhibition of DBH could be a potential reason for this phenomenon.

Dysregulation of the mesocorticolimbic circuit leads to social deficits, and malfunction of the nigrostriatal circuit leads to stereotyped behaviors seen in ASD (17). Thus, the corpus striatum can be considered a critical area relevant to ASD as it receives projections from both systems. Therefore, CDI mediated alterations in the striatum should be further explored as a risk factor for ASD in infants and in patients with existing ASD.

### C. difficile induced alterations in catecholamine profiles in hippocampus and substantia nigra.

**FIG 4:**
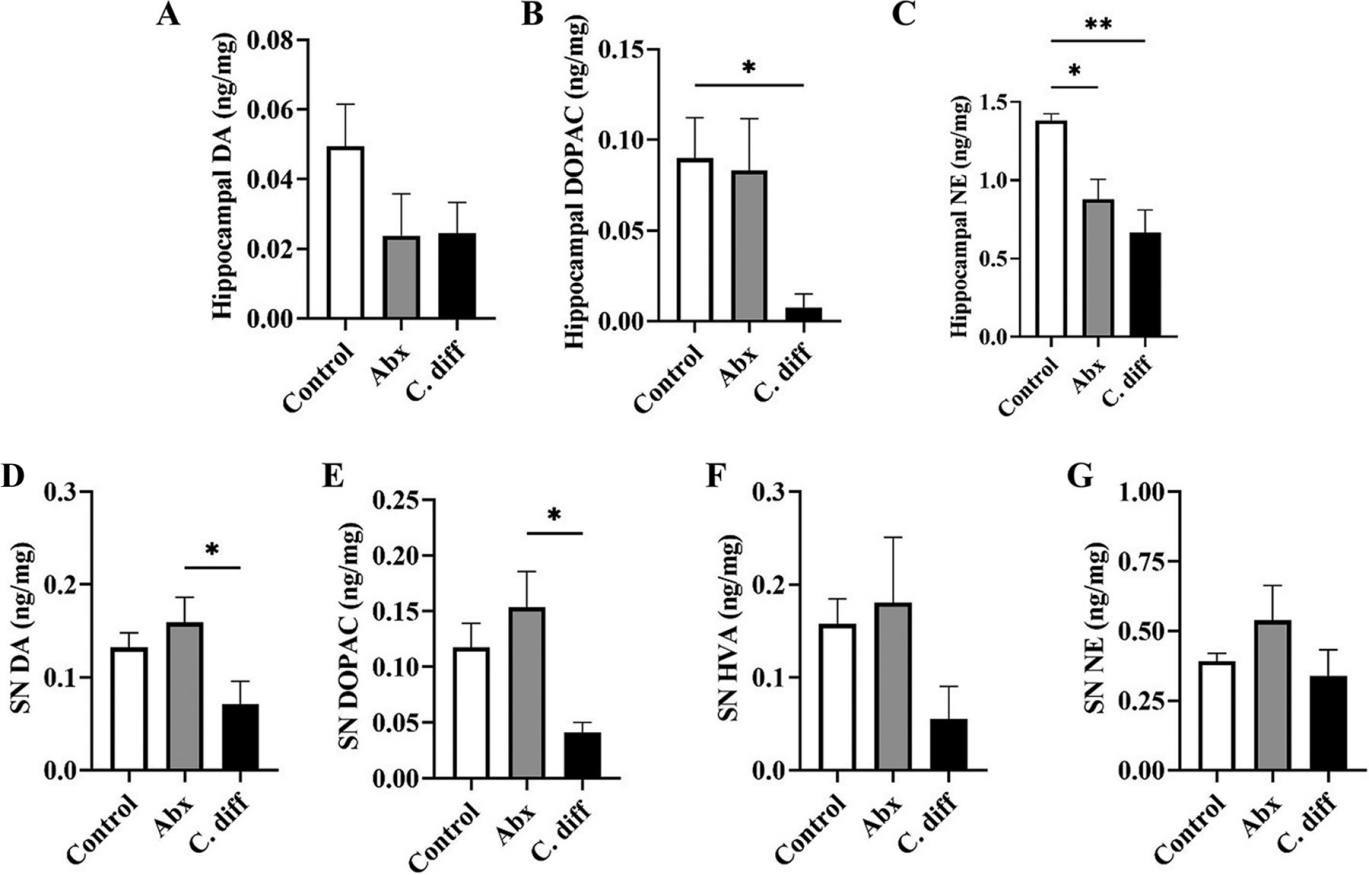
Alterations in hippocampus and substantia nigra catecholamine profiles in antibiotic treated and C. difficile infected mice. Hippocampal catecholamine concentration in different treatment groups: (A) dopamine (DA), (B) its metabolite 3, 4-dihydroxyphenylacetic acid (DOPAC), and (C) norepinephrine (NE) levels. (Hippocampal HVA levels were below the detection limit in all groups); substantia nigra (SN) catecholamine concentration in different treatment groups: (D) dopamine (DA), (E) its metabolite 3,4-dihydroxyphenylacetic acid (DOPAC), (F) homovanillic acid (HVA), and (G) norepinephrine (NE) levels. *n* = 5. All data (A–G) are presented as the mean, and error bars indicate SEM. One-way ANOVA with adjusted *P*-value was used to test for statistical significance **P < 0.01, *P < 0.05.

In contrast, the hippocampal neurochemical profile revealed an unaltered or decreasing trend of DA and DA degradation products in C. difficile infected animals (Fig. 4A to C). This may suggest the dominance of the pain and memory-associated nondopaminergic pathways in the hippocampus in response to CDI. Interestingly, a significant decrease in NE is noticed in the hippocampus, suggesting a reduced production of the NE in the nor-adrenergic neurons projecting to the hippocampus (Fig. 4C). This enables exploration into β-noradrenergic-mediated hippocampal-basolateral amygdalar control of social recognition memory consolidation, which is believed to be disordered in individuals with ASD and therefore may have difficulty to establish a social relationship and social groups (21). In addition, we examined the catecholamine profile in one of the two major dopamine producing areas in the brain that projects to the striatum, i.e., substantia nigra (Fig. 4D–G). In addition to an unaltered NE level, decreased concentrations of DA (*P* = 0.0379) and DOPAC (*P* = 0.0135) are observed in infected animals, suggesting a potential feedback inhibition of DA production in response to the accumulation of DA in the striatum (Fig. 4D, E, G) (22). This is also in line with the hypothesis of altered DA to NE conversion in the brain in response to inhibition of DBH.

### C. difficile induced alterations in serum DBH activity.

**FIG 5:**
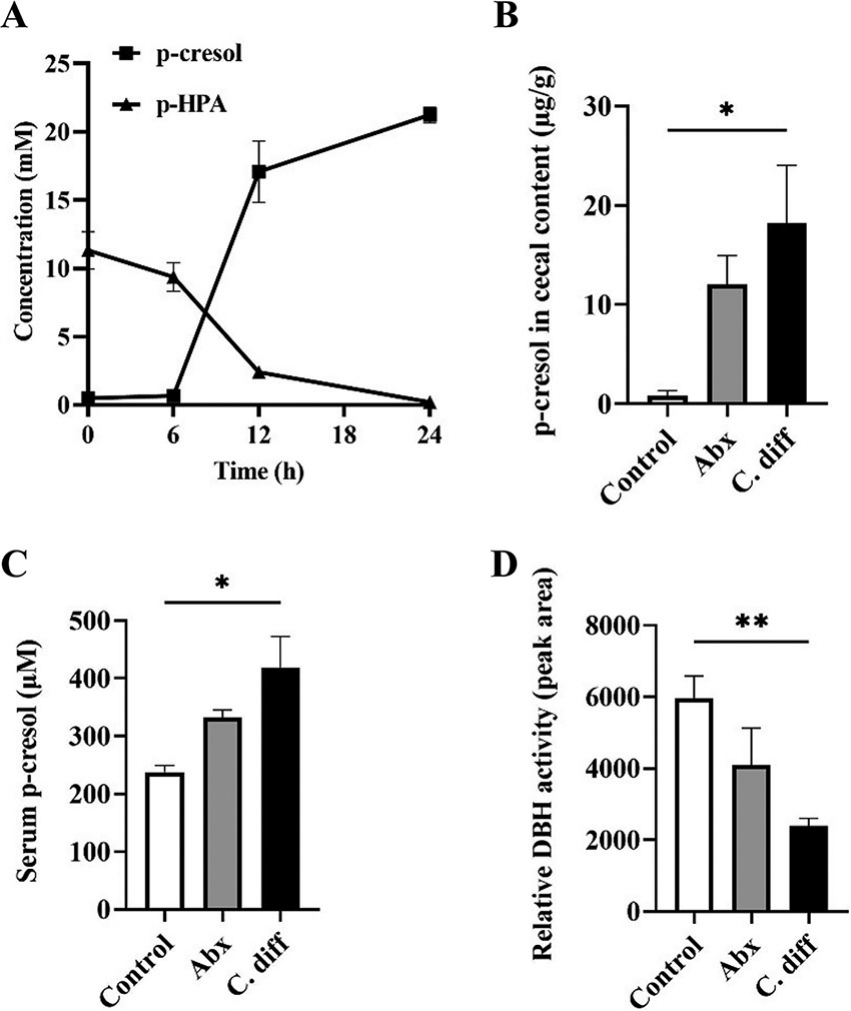
C. difficile infection in mice increases cecal and serum p-cresol levels and reduces serum DBH enzyme activity. (A) p-cresol producing ability of C. difficile UK1: C. difficile *UK1* converted the substrate p-HPA to p-cresol in 24 h (*n* = 3). (B) Cecal p-cresol concentration: C. difficile infection significantly increased cecal p-cresol levels in mice (*n* = 4). (C) Serum p-cresol concentration: C. difficile infection significantly increased serum p-cresol levels (*n* = 4). (D) Relative serum DBH activity: C. difficile infection significantly decreased serum DBH activity as indicated by a reduced peak area representing the tyramine octopamine transformation per hour (*n* = 4). All data in (A–D) are presented as the mean, and error bars indicate SEM. One-way ANOVA with adjusted *P*-value was used to test for statistical significance **P < 0.01, *P < 0.05.

The enzymatic assay performed on the serum samples of C. difficile infected and control mice confirmed a significant reduction in the DBH activity (*P* = 0.0099) (Fig. 5D). Minor increases in the serum DBH (not significant) were observed in the Abx group compared to the control group. Notably, a low maternal serum level of DBH and mutations in the DBH genes have been associated with autism in children (19, 20). The brain is the primary source of the serum levels of DBH, and either serum or plasma DBH activity has been considered one of the reliable measures of brain DBH activity (23). It has been previously established that p-cresol, a toxic metabolite produced by C. difficile is a potent inhibitor of this enzyme (24). DBH catalyzes the conversion of DA to NE in dopaminergic and noradrenergic neurons, and inhibition of DBH can increase the DA levels in the neurons and synapses with a concurrent reduction in the NE levels (23, 24). The neurochemical profile of the striatum and prefrontal cortex of the C. difficile challenged mice showed significantly increased DA or DA degradation product in these brain areas, suggesting that these alterations could be solely or partially a result of p-cresol mediated inhibition of DBH enzyme.

### C. difficile induced alterations in intestinal and serum p-cresol levels.

This study used a p-cresol producing human hypervirulent C. difficile strain (UK1). To confirm the p-cresol producing ability of C. difficile UK1, a p-HPA-p-cresol conversion assay was performed. Our results indicated that C. difficile UK1 completely metabolized the substrate p-HPA to p-cresol in brain heart infusion (BHI) media by 24h of incubation in anaerobic conditions (Fig. 5A), confirming this particular property of C. difficile.

One of the major results from this study shows that CDI induces an increase in serum p-cresol (*P* = 0.0199) (Fig. 5C), in addition to an expected increase in intestinal p-cresol levels (*P* = 0.0240) (Fig. 5B), which may have direct implications in the pathogenesis and aggravation of ASD-like behavior in autistic patients. An increasing trend in the cecal and serum p-cresol, although not statistically significant, was observed in the Abx group compared to control. Human C. difficile strains produce a significant amount of p-cresol, unlike most other gut bacteria (25). C. difficile produces 10 to 1000 times more p-cresol than other known p-cresol producing bacteria in the gut (25). p-Cresol produced by gut bacteria crosses the blood brain barrier and has been detected in higher levels in cerebrospinal fluid of patients in Parkinson's disease, a disorder of the dopaminergic system (25, 26). Moreover, it is already known that patients with ASD often have a significantly higher urine p-cresol level, which is considered a biomarker for this disease complex (27).

A recent study by Pascucci et al. (2020) demonstrated that intravenous p-cresol injection aggravates autistic behaviors in BTBR (Black and Tan Brachyury) mice, a murine genetic model of ASD (11). Similar results are also demonstrated following oral p-cresol administration in nonmutant mice by Bermudaz-Martin et al. (2021) (12). In Pascucci's study, a single short-term intravenous p-cresol administration aggravated autistic behavior in mice and most notably, increased the striatal DA levels. Interestingly, the serum p-cresol assay results and striatal and hippocampal neurochemical profile from C. difficile infected mice corroborate Pascucci et al.'s observation. This study also suggested potential involvement of p-cresol mediated DBH enzyme inhibition to explain this observation.

Similarly, Bermudaz-Martin et al. (2021) reported impaired excitability of dopaminergic neurons and precipitation of autistic behavior in C57BL/6 mice continuously administered with oral p-cresol in drinking water (12). In contrast, Bermudaz-Martin et al. (2021) attributed p-cresol mediated behavioral and neuronal alterations to remodeling of gut microbiota (gut dysbiosis) mediated by p-cresol, by demonstrating similar changes in mice transplanted with fecal material from p-cresol administered animals. However, Pascucci's results indirectly nullify this theory by demonstrating similar behavioral and neurochemical alterations in the short term, intravenous administration of p-cresol in BTBR, which entirely bypasses the involvement of gut and leaves minimal opportunity for dysbiosis or microbial remodeling. Thus, an alternate but direct mechanistic pathway, such as inhibition of DBH, as proposed by Pascucci et al. and supported by our current study, could be a plausible explanation for p-cresol induced alterations in dopaminergic neurotransmission.

p-Cresol-producing bacteria, such as C. difficile, are extremely resistant to high gut cresol levels. Moreover, p-cresol strongly inhibits the growth of other bacteria in the gut, providing a selective advantage for p-cresol-producing bacteria to overgrow (25). This may result in further enhanced p-cresol production in the gut and p-cresol mediated neurologic alterations in mice transplanted with such gut content, which more reasonably explains Bermudez-Martin's observation. Regardless, Bermudez-Martin's experiment complements Pascucci et al.'s study in several ways, as the more prolonged nature of p-cresol administration in their study resulted in DA receptor insensitivity. It has been previously reported that prolonged, high DA levels increase dopaminergic stimulation and impair DA receptor sensitivity in postsynaptic neurons (28), supporting the results described in the current study and Pascucci et al.'s report. Therefore, the results from this study suggest a potential link between antibiotic-induced CDI, bacterial p-cresol production, reduced DBH activity, and an altered dopaminergic axis, supporting the previous reports of p-cresol mediated ASD-like behavioral alterations in rodents.

These observations are particularly important in infants with antibiotic-associated C. difficile overgrowth, predominantly asymptomatic in infants (29). A high early postnatal DA level in the rapidly developing brain may permanently alter the dopaminergic axis and dopamine receptor sensitivity, which may have a contributory role in precipitating regressive autism. Thus, the current study suggests a plausible link between C. difficile infection and precipitation or aggravation of ASD in infants.

About one-third of healthy human infants are colonized with C. difficile in their gut, and this bacterial population disappears typically during the weaning period (30, 31). C. difficile colonization in infants is usually asymptomatic and does not cause diarrhea despite presence of C. difficile exotoxins in their stool (31–34). Antibiotic therapy and/or disruption of the gut microbiome (dysbiosis) induce C. difficile overgrowth, which manifests as enterotoxin-mediated diarrhea, especially in adults (35). Interestingly, toxin-mediated diarrhea is generally absent in infants due to unique immunologic peculiarities of the infant gut as well as toxin binding molecules present in breast milk (29, 36). Thus, C. difficile overgrowth in human infants is likely less noticeable clinically compared to adults. Although causation has not yet been proven, recent observations suggest a significant association between antibiotic exposure in early infancy and autism (37, 38) (mostly to treat ear infections; interestingly, children with ASD have higher incidents of otitis media [39]). For example, a recent study reported a significantly increased risk of autism after exposure to cephalosporin antibiotics (38). Nonetheless, infants fed commercial formula are documented to have a higher C. difficile colonization in their gut and are overrepresented in ASD patients compared to breastfed children (40, 41). These published observations suggest an association between antibiotic therapy, gut-dysbiosis, and regressive autism in children, where CDI should be investigated as a potential additional risk factor.

In summary, the current study demonstrated that antibiotic-induced CDI causes significant alterations in the dopaminergic axis of mice. In addition, CDI significantly increased circulating p-cresol levels and reduced DBH activity in infected animals. Thus, the published studies, together with our current observations, suggest a potential link between CDI, gut and serum p-cresol, DBH activity, and alterations in dopaminergic neurotransmission that may have direct implications in the precipitation and aggravation of neurodevelopmental disorders.

## MATERIALS AND METHODS

### Bacterial culture, media, and in vitro p-cresol production assay.

The human epidemic C. difficile strain, UK1 (ribotype 027), was used in this study. C. difficile was cultured on two prereduced media: (i) BHI broth supplemented with yeast, (ii) BHI broth supplemented with yeast and p-HPA, in anaerobic conditions (0% oxygen, 5% hydrogen, 5% CO_2_, and 90% nitrogen at 37°C) and incubated in an anaerobic workstation (AS-580, Anaerobe Systems, Morgan Hill, CA, USA). Primary C. difficile cultures were grown on BHI broth for 13 h and then were diluted 1/100 into pre-equilibrated media with and without 0.3% p-HPA. After 24 h, culture supernatant was collected after centrifugation at 5,000 × *g* at 4°C for 15 min, followed by filter sterilization. Supernatants were immediately aliquoted and saved at −80°C until analysis. All experiments were run in triplicates using prereduced media in an anaerobic environment, with every experiment being repeated twice. p-Cresol and p-HPA concentrations in the culture supernatant were estimated using the HPLC method as per standard protocol (42).

### Mouse model for antibiotic-induced C. difficile infection.

All animal experiments were performed as per the protocols approved by ISU Institutional Animal Care and Use Committee (IACUC-20-091). Fifty-four C57BL/6 mice (3 to 4 weeks old) with an equal male-female ratio were purchased and housed in groups of 2 per cage with sterile food, water, and bedding. Mice were randomly assigned to each of the three groups: Control, antibiotic control (Abx), and antibiotic induced C. difficile infection (C. diff) groups. After 4 days of acclimatization, Abx and C. diff groups received an antibiotic mixture in drinking water (kanamycin,0.4 mg/mL, gentamicin,0.03 mg/mL, colistin,850 U/mL, metronidazole, 0.215 mg/mL, and vancomycin,0.045 mg/mL) for 4 days. After the antibiotic treatment, the mice were given regular autoclaved water for 1 day, and all animals in the Abx and C. diff groups received a single dose of clindamycin (10 mg/kg, maximum volume of injection 0.5 mL/mouse using a 27-gauge gavage needle and syringe) intraperitoneally 1 day before *C. difficile* challenge. This antibiotic pretreatment was intended to disrupt the normal gut flora of mice and facilitate C. difficile colonization (13). Further, the C. diff group mice were orally gavaged with 100 μL of 1.4 × 10^4^ C. difficile spores (13). Respective control animals in the experiment were administered with sterile PBS as a sham in each step accordingly. The animals were euthanized using carbon dioxide gas at approximately 48 h postinoculation, and the cecal contents and intestinal tissue samples, serum, and brain sections were collected for further processing.

### Histopathology.

The cecum and colon tissues were fixed in 10% formalin and then embedded in paraffin. A 5 μm thickness of tissue sections was made and stained with hematoxylin and eosin. A standardized scoring system was followed to assess CDI-associated histology injuries in intestinal tissues, and scores were evaluated across the groups based on epithelial damage, mucosal edema, and neutrophil infiltration (43). A board-certified pathologist (sample ID-blinded) performed microscopic analysis using an Olympus BX53 microscope (Olympus Optical Company, Tokyo, Japan). Scores were combined into a cumulative histologic score for each mouse ranging from 0 to 9, with 0 being no epithelial damage/mucosal edema/neutrophil infiltration in the sample and a score of 9 vice versa.

### Microbial DNA extraction and sequencing.

Fecal metagenomic DNA was extracted using the DNeasy PowerSoil Pro kit (Qiagen, Germantown, MD) automated for high throughput on QiaCube HT (Qiagen, Germantown, MD), with bead beating in Qiagen Powerbead Pro plates (Cat No. 19311). Samples were quantified with Qiant-iT Picogreen dsDNA assay kit (Invitrogen, Waltham, MA). The extraction and sequencing steps were performed by Diversigen (New Brighton, MN). Libraries were prepared with a procedure adapted from the Nextera Library Prep kit (Illumina, San Deigo, CA). Libraries were sequenced on an Illumina NovaSeq using single-end 1 × 100 reads (Illumina, San Diego, CA).

### Metagenomic analysis.

Reads were annotated using the BoosterShot in-house pipeline (Diversigen, New Brighton, Minnesota), as follows as per previously published protocol (44, 45). DNA sequences were filtered for low quality (Q-Score < 30 bp) and length (<50 bp), and adapter sequences were trimmed using cutadapt. Reads were aligned to the host genome using Bowtie2, a minimum alignment score of [-0.6 + -0.6 * L] where L is the read length. Only reads not aligning to the host were carried forward. Sequences were trimmed to a maximum length of 100 bp prior to alignment. DNA sequences were aligned to a curated database containing all representative genomes in RefSeq for bacteria with additional manually curated strains. Alignments were made at 97% identity against all reference genomes. Every input sequence was compared to every reference sequence in Diversigen's Venti database using fully gapped alignment with BURST optimal aligner (46, 47). Ties were broken by minimizing the overall number of unique Operational Taxonomic Units (OTUs). For taxonomy assignment, each input sequence was assigned the lowest common ancestor that was consistent across at least 80% of all reference sequences tied for the best hit. The number of counts for each OTU was normalized to the average genome length. OTUs accounting for less than one millionth of all species-level markers, and those with less than 0.01% of their unique genome regions covered (and < 1% of the whole genome) were discarded. Samples with fewer than 10,000 sequences were also discarded. Count data were then converted to relative abundance for each sample. The normalized and filtered tables were used for all downstream analyses. Kyoto Encyclopedia of Genes and Genomes Orthology groups (KEGG KOs) were observed directly using alignment at 97% identity against a gene database derived from the strain database used above. The KO table and downstream tables contain the directly observed KO counts converted to relative abundance within a sample. KOs were collapsed to level-2 and -3 KEGG pathways and KEGG modules. Bray-Curtis beta diversity metrics were calculated from the filtered species table and the KEGG module and enzyme relative abundance tables using QIIME 1.9.1 (48). The Chao1 index, Shannon index, and observed OTU count (taxonomic group) were calculated using a rarefied OTU table set to the minimum depth allowed for a sample (10,000) using QIIME 1.9.1.

### HPLC-ECD for tissue catecholamine analysis.

Mouse brain regions, including striatum, prefrontal cortex, substantia nigra, and hippocampus were micro-dissected with mouse brain matrix (ASI Instruments, RBM-1000S), preserved immediately in an antioxidant mixture containing 0.2 M perchloric acid, 0.1% Na_2_S_2_O_5_, 0.05% Na-EDTA, and isoproterenol (internal standard) as described previously and directly stored in dry ice (49). The samples were homogenized, sonicated, and filtered in 0.2-μm filter tubes. 20 μL samples were injected into High-Performance Liquid Chromatography – Electrochemical Detector (HPLC-ECD) with a pump (Thermo-Scientific ISO-3100SD-BM), autosampler (Thermo-Scientific WPS-3000 TSL), and CoulArray 5600A-ECD detection system operated at 23°C connected to Agilent Eclipse Plus (3.5 μm, 100A) C18 HPLC column (150 × 4.60 mm). Tissue neurochemical analytes were eluted in MD-TM mobile phase (cat. no. 701332, Thermo Fisher Scientific) under isocratic conditions at 0.6 mL/min for 21 min. Dopamine (DA) and its major metabolites, Norepinephrine, 3,4-dihydroxyphenylacetic acid (DOPAC), and homovanillic acid (HVA), were analyzed using CoulArray Data Station (v. 3.10) and quantified using a standard curve. Tissue neurochemical concentrations were normalized using wet tissue weight (mg) and corrected using the internal standard extraction coefficient.

### Quantification of p-cresol.

Blood was collected by cardiac puncture. Serum was separated following clotting and centrifugation at 2500 × *g* for 15 min. The collected serum was aliquoted as 40 μL into multiple microcentrifuge tubes and immediately temporarily stored in dry ice and further long-term storage at −80°C until analysis. The cecal contents were collected and snap-frozen using liquid nitrogen and stored at −80°C until quantitative analysis via mass spectrometry.

**(i) Reagents.** 4-ethyl phenol, p-cresol (pC), salicylic acid (S.A.) were purchased from Millipore-Sigma (St. Louis, MO), and p-cresol sulfate (pCS) and p-cresol glucuronide (pCG) were purchased from United States Biological, (Salem, MA). All the solvents were LC-MS grade and purchased from Fisher Chemical unless otherwise mentioned.

**(ii) Instrumentation.** Conjugated cresols were analyzed via an Agilent 6470 triple quadrupole (QQQ) mass spectrometer with electrospray ionization equipped with an Agilent 1290 Infinity II UHPLC (Agilent Inc, MA, USA). Conjugated cresols were separated with the Agilent C18 column (2.1 × 100 mm, 1.8 μm) at 40°C. The mobile phase consisted of A: 5 mM ammonium acetate + 0.005% acetic acid and B: acetonitrile with a 0.400 mL/min flow rate. The solvent gradient was maintained at 0 min, 0% B; 0–0.25 min, 0–100% B; 0.5–15 min, 100–0% B; 15–20 min, with a 3-min pre-equilibration.

**(iii) Quantification.** Analysis was performed in negative mode with the nozzle voltage set at 2000 V. Nitrogen was used as both nebulizer, desolvation, and sheath gas. The nebulizer pressure was set at 40 lb/in^2^ with a capillary voltage of 2500V. Desolvation gas was delivered at 12 L/min with temperature heated to 350°C. The sheath gas flow was 10 L/min at a temperature of 350°C. High-purity nitrogen was used as a collision gas for collision induced dissociation. Multiple reaction monitoring was used to detect cresol conjugates, the precursor, product ions, and collision energies are as follows: pCG precursor *m/z* 283 product *m/z* 107, 18 eV; pCS precursor *m/z* 187 product *m/z* 107, 18 eV; S.A. precursor *m/z* 137 product *m/z* 93, 25 eV.

Free p-cresol was analyzed with an Agilent 5975C quadrupole mass spectrometer equipped with 7890A gas chromatograph. The capillary column used was HP-88 (60m x 0.25 mm x 0.2 μm). An injection volume of 2 μL was used with the inlet operating in splitless mode. The oven GC program used an initial temperature of 40°C for 1 min, then increased to 230°C at a 20°C/min rate, with a final hold of 7.5 min. Inlet and mass-selective detection transfer line temperatures were held at 250°C. Mass detection was conducted under standard settings with a mass detection range set to *m/z* 40–600. Peak identification was performed using Qualitative Analysis (version 10.0) software and the NIST Mass Spectral Search Program (version 2.3) with the NIST17 and Wiley 11 GC-MS spectral library. Peak detection and integration were accomplished using Agilent MassHunter Quantitative Analysis (version 10.0) software to monitor *m/z* 107 and *m/z* 108.

**(iv) Sample preparation for conjugated p-cresol.** The extraction protocol was adapted from Morinaga et al. (2004) and Shu et al. (2016) with modifications as described here (50, 51). The analysis of p-cresol conjugates was completed starting with 25–100μL serum samples or 150–500 mg gut contents previously archived in microcentrifuge tubes. Samples were kept on ice throughout the extraction. 4-ethyl phenol and salicylic acid were used as internal standards. These two internal control samples were spiked into the samples as follows: 4-ethyl phenol, 10 μL of 0.25 mg/mL in ethyl acetate (2.5 μg); salicylic acid, 10 μL of 0.05 mg/mL in 50% methanol (0.5 μg). These internal standards were also spiked into 200 μL of 0.9% NaCl spiked and were used as a negative control. Three volumes (300 μL/100 mg-sample) of extraction solvent mixture of acetonitrile and water (2:1 vol/vol) were mixed with the sample, and after a quick vortex, this was incubated on ice for 10 min. The samples were sonicated for 10 min followed by a 10 min vortex. The supernatant was recovered after 7 min centrifugation at 13,000g. Pellets were saved in the −20°C freezer for free p-cresol extraction. 200 μL of the supernatant was filtered through a spin filter (catalog number UFC30LG25, Merck Millipore). Samples were subjected to LC-MS/MS analysis, 5 μL of which were directly injected into the LC-MS (QQQ) (51).

**(v) Sample preparation for free p-cresol.** Analysis of free p-cresols was done with a continuation of the above extract for conjugate cresols. The supernatants were recombined with their respective pellets in the microcentrifuge tubes. Two volumes (200 μL/100 mg-sample) of 0.9% NaCl were added to the mix, followed by the addition of a solvent mix of 1:1 hexane: ethyl acetate (200 μL). The mixture was sonicated for 10 min, followed by 10 min of vortexing, and finally centrifuged for 7 min to obtain the phase separation. The top layer, which contains free cresols, was carefully transferred into a GCMS vial.

**(vi) Preparation of calibration curves.** Standards were made for conjugates in 2:1 acetonitrile:water 0.1–0.0005 mg/mL, and for p-cresol in ethyl acetate 0.75–0.0015 mg/mL. 10 μL of these standards were added in to 200 μL 0.9% NaCl. Internal standards were also added and extracted by following the same extraction procedure for the samples.

### Dopamine Beta-hydroxylase (DBH) activity assay.

*Chemicals and reagents:* All assay chemicals were purchased from Millipore-Sigma unless otherwise stated. All solvents were LC-MS grade and purchased from Fisher Chemical.

**(i) Sample preparation and DBH assay reaction.** Serum samples (40 μL) were prepared and assayed as previously described by Punchaichira et al., 2018, with the addition of LC-MS quantification as described below (52–54). Briefly, 40 μL of the serum samples were added to a cocktail of 1.0 mM tyramine HCl, 10 mM fumarate, 0.1 mg/mL catalase, 4 mM ascorbate, 2 μM copper sulfate, 30 mM N-ethylmaleimide and 1.0 mM pargyline in 125 mM sodium acetate pH 5.2. The mixture was incubated for 1 h at 37°C followed by the addition of 50 μL of 25 mM EDTA for stopping the reaction.

**(ii) Instrumentation.** Equipment included Agilent Technologies 1100 Series HPLC system coupled to both a UV-Vis capable diode array detector (DAD) and an Agilent Technologies Mass Selective Trap SL detector equipped with a SphereClone 5 μm ODS (2) 80 A, L.C. Column 250 × 4.6 mm (Phenomenex) held at 40°C, and an electrospray ion source operated in positive mode. Running solvents consisted of A: 0.1% acetic acid in water and B: 0.1% acetic acid in acetonitrile. The flow rate was 0.700 mL/min with isocratic conditions of 35% B. The ion source drying gas was nitrogen which was set to flow at 12 mL/min at 350°C, the nebulizing gas was set to 30 lb/in^2^. The mass scan range was *m/z* 75–300 with a max accumulation time of 300 ms.

**(iii) Quantification of enzyme activity.** To quantify DBH activity, 10 μL of each prepared serum DBH assay sample was injected for LC-MS analysis. The amount of octopamine converted from tyramine was quantified by monitoring the MS/MS product ion *m/z* 119 fragmented from the [M+H]^+^ ion (*m/z* 154) of octopamine at 0.7 fragmentation amplitude. An octopamine standard curve was generated in assay buffer ranging from 0–2 nmol-octopamine/sample. Quant analysis for 6300 Series Ion Trap LC/MS Version 1.8 (Bruker Daltonik, Bremen, Germany) software was used for the LC-MS/MS data analysis.

### Statistical analysis.

Statistical analysis was performed using GraphPad Prism 9 (GraphPad Software, San Diego, CA) with *P* < 0.05 considered statistically significant. All results were expressed as means ± standard errors of the means (SEM) unless otherwise indicated. The differences between the experimental groups were compared using the analysis of variance (ANOVA).
